# Comparison of monocytic cell lines U937 and THP-1 as macrophage models for in vitro studies

**DOI:** 10.1016/j.bbrep.2022.101383

**Published:** 2022-11-18

**Authors:** Camyla Rodrigues Nascimento, Natalie Ap Rodrigues Fernandes, Laura Andrea Gonzalez Maldonado, Carlos Rossa Junior

**Affiliations:** a Department of Diagnosis and Surgery, School of Dentistry at Araraquara, São Paulo State University (UNESP), Araraquara, SP, Brazil; b Department of Periodontics and Oral Medicine, School of Dentistry, University of Michigan, Ann Arbor, MI, USA

**Keywords:** Macrophage, Gene expression, THP-1 cells, U937 cells, Phenotype

## Abstract

Understanding macrophage biology can improve comprehension of diverse biological processes and provide insights into novel therapeutic immunomodulatory strategies. Due to limited yield and technical difficulty in isolating primary macrophages, in vitro studies commonly use monocytes as precursor cells. Monocytic cell lines are a virtually unlimited source of macrophage precursors and two of the most frequently used cell lines are THP-1 and U937. Besides a great variability in macrophage differentiation protocols there is scarce information on possible differences in the biological responses of these cell lines. In this study, we used a standardized differentiation protocol using PMA and compared the response of macrophages derived from THP-1 and U937 cells to M1-and M2-polarizing conditions. THP-1-derived macrophages are more responsive to M1 stimuli and skewed towards M1 phenotype, whereas U937-derived macrophages were more responsive to M2 stimuli and skewed towards M2 phenotype. THP-1-derived macrophages also had greater production of ROS and phagocytic activity. Under M1-polarizing conditions, macrophages derived from both THP-1 and U937 reduced phagocytosis activity and the increased production of ROS. This information should be considered to make an informed choice on the cell line used as in vitro macrophage model, according to the experimental goals and biological context.

## Highlights

• THP-1 and U937 were successfully differentiated in macrophages using 10 ng/mL of PMA.
• M-CSF did not promote macrophage differentiation of either THP-1 or U937 cells.
• THP-1 cells are skewed to the M1 phenotype.
• U937 cells are skewed to the M2 phenotype.

## 1 Introduction

Macrophages in inflammatory conditions and cancer frequently originate from circulating monocytes [19]. Macrophages are responsive to various microenvironmental signals (including Microbe or Damage Associated Molecular Patterns- MAMPs and DAMPs, respectively) that modulate their activation status and phenotype. Diverse biological mediators (chemokines, cytokines, metalloproteinase, products of enzymatic reactions such as prostaglandins, leukotrienes, reactive oxygen species- ROS, and others) produced by macrophages can influence the afflux and activation of other cell types in the microenvironment, further shaping the host response and ultimately reestablishing homeostasis [12].

The relevance of macrophages in various diseases, conditions and repair processes justifies the great interest in these cells, since understanding the biology of macrophages and the possible mechanisms by which they can modulate pathogenesis and repair processes can be explored for the development of new therapeutic approaches [10,20]. In vitro studies using human cells are critical in investigating macrophage biology and rely either on primary cells obtained from donors or on established monocytic cell lines [3].

Peripheral blood mononuclear cells (PBMC) are isolated from whole blood and include T and B lymphocytes, monocytes, and dendritic cells. The maintenance of primary cells in culture is usually more demanding compared to established cell lines. The yield of the primary monocyte isolation process is variable, since the proportion of this cell type in PBMC range from 2 to 10%, thus availability is limited compared to established monocytic cell lines. Moreover, there may be important inter- and intra-individual variability in phenotype and cellular response in different individuals [11,26].

The use of pre-established monocytic cell lines provides virtually unlimited availability, reduced cost, and greater consistency of biological response. THP-1, U937, MonoMac 6, ML-2 and HL-60 are frequently used human monocytic cell lines, which have in common unlimited proliferation and resistance to apoptosis because of their neoplastic (leukemia) nature. Thus, the convenient characteristics of these cell lines also represent their main limitation, since the biological response of neoplastic cells can be substantially different from that of primary non-neoplastic cells [7]. Despite this limitation, monocytic cell lines are intensely used because they allow the investigation of relevant biological mechanisms, which are subsequently verified in in vivo models.

THP-1 and U937 are the most commonly monocytic cell lines used as macrophage precursors because of their ability to mimic the macrophage differentiation process and for their similarities with primary monocytes and macrophages in morphology and biological responses [1]. THP-1 cells can be transfected, expresses C3 complement, Fc receptors, have phagocytic activity and can be differentiated into macrophages by phorbol esters (TPA or PMA) [6]. U937 cells can also be transfected and express C3 complement in addition to TNF-α and can be differentiated into macrophages by phorbol esters (PMA). Differences between these cell lines include their origin, as U937 cells are derived from histiocytic lymphoma (male patient, 37 years old), whereas THP-1 cells are derived from acute monocytic leukemia (male patient, 1 year old); the fact that U937 cells are more mature and immortalized by transformation by Epstein-Barr virus, whereas THP-1 cells are spontaneously immortalized. Both THP-1 and U937 cells are not demanding in their culture conditions [23,36].

Despite the limitations inherent to in vitro studies, the use of these cell lines has yielded important and replicable information that contributed to the advancement of knowledge on macrophage biology and their relevance in diverse contexts [5,31]. Nevertheless, there are indications of important differences in the biological response of THP-1 and U937 cells, and the choice of one or the other lineage is often not biologically justified in the published studies. Speculatively, the use of U937 or THP-1 cells may be primarily related to the availability and/or personal experience of the investigators. Thus, identifying possible differences in cellular response of these cell lines and characterizing biological aspects involved in macrophage differentiation and polarization in vitro can aid researchers to select the most appropriate cell line for the specific purpose of the study and experimental model.

There is no consensus on the protocols used for their differentiation and polarization, making it difficult to interpret conflicting results reported in the literature. This study will explore the biological behavior of these cell lines in response to a pre-defined and standardized differentiation protocol and polarization conditions, generating information that is useful to several areas of study using these cell lines as in vitro models of macrophages.

## 2 Material and methods

### 2.1 Cell lines

Human monocytic cell lines THP-1 and U937 were obtained from ECACC and ATCC, respectively, both certified as mycoplasma-free. THP-1 and U937 cells were cultured in Roswell Park Memorial Institute (RPMI)-1640 medium supplemented with 10% heat-inactivated fetal bovine serum (FBS), 1% antibiotics (P/S-penicillin and streptomycin) in a humidified incubator at 37 °C with 5% CO_2_, as indicated by the suppliers. Mycoplasma contamination was regularly checked in aliquots of cells differentiated onto coverslips and fixed in 4% paraformaldehyde by direct DNA staining with DAPI and observation on an inverted fluorescence microscope (Evos *fl*, AMG Micro) [24]).

### 2.2 Differentiation of THP-1 and U937 cells in macrophages with PMA and M-CSF – morphological and cytoskeletal changes

Recombinant human (rh) M-CSF (Peprotech Inc.) was used as a macrophage differentiation stimulus at 50 and 100 ng/mL rhM-CSF stimulation was carried out in complete (10% FBS, 1% P/S) RPMI medium for a total of 6 days, with refreshment of 50% of the volume of M-CSF-supplemented complete culture medium on the third day. At the end of the 6-day differentiation period, cells attached to the culture substrate were considered as ‘unstimulated macrophages’.

**Fig. 1:**
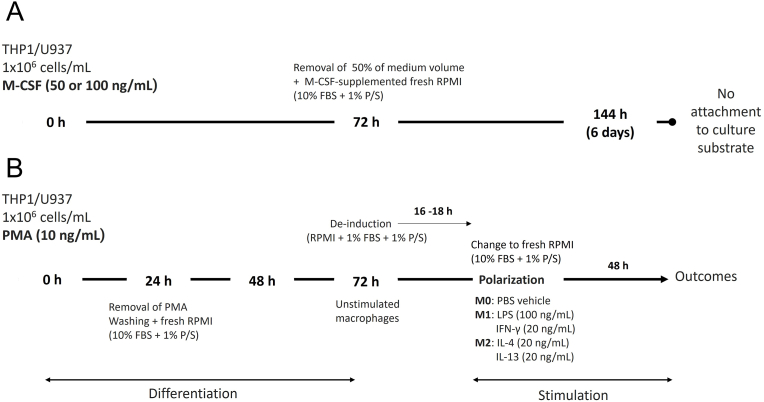
Timeline of the experimental protocol used for M-CSF and PMA-induced macrophage differentiation and subsequent polarization in M1 and M2 conditions.

Phorbol-Myristil-Acetate (PMA) was the other macrophage differentiation stimulus used. There is a plethora of differentiation protocols reported in the literature, with variations in both PMA concentration and time of treatment. We used one defined protocol to allow for comparisons between the biological response of the monocytic cell lines. We chose a recently described [4] differentiation protocol that is shown to be conducive to näive (i.e., poorly activated) macrophages. Except for the assessment of morphology and cytoskeleton in which 7 × 10^4^ cells were differentiated in chamber slides, a total 1 × 10^6^ monocytic cells in 1 mL of complete culture medium were treated with 10 ng/mL of PMA for 24 h, the medium containing PMA was then removed, and cells washed gently with PBS to remove residual PMA and non-adhered/dead cells. Complete RPMI medium (10% FBS, 1% P/S) was added, and cells were maintained in culture for an additional resting period 48 h. At the end of this period, adhered cells were considered as ‘unstimulated macrophages’. Fig. 1 presents the timeline of the experimental protocol, including macrophage differentiation and stimulation/polarization of the macrophages. Overall, when PMA was used, monocytic cells were differentiated over a period of 72 h and subsequently polarized/stimulated over a period of 48 h.

### 2.3 Morphology

Monocytic cells were cultured in 4-well chamber slides at a density of 70,000 cells/mL (500 μL/well). At the end of the differentiation period (Figs. 1–24 h and 72 h for PMA, 6 days for M-CSF) the morphology of cells was observed on a digital inverted microscope (brightfield). Changes in cell area and perimeter were assessed using ImageJ software (http://imagej.nih.gov/ij) in all cells present in brightfield digital images of three random microscopic fields per well in three independent experiments performed in duplicate. The assessments of cell area and perimeter were performed by a trained operator who was unaware of the cell line, experimental period and method of differentiation used.

### 2.4 Cytoskeleton

Immediately after imaging cells in brightfield in each period (24 and 72 h for PMA, 6 days for rhM-CSF), cells were fixed with 2% paraformaldehyde, permeabilized in saponin-containing buffer and stained with AlexaFluor 488-conjugated phalloidin (Thermo Fisher Scientific) (40 nM) for 40 min, followed by DNA staining with DAPI (Thermo Fisher Scientific) (2 μg/mL) for 5 min. After extensive washing with PBS, images were obtained at 100 and 400X magnification on an inverted digital fluorescence microscope (Evos *fl*, AMG Micro) in three random fields for each coverslip.

### 2.5 Phenotypical polarization

Since rh-MSCF did not induce macrophage differentiation of either THP-1 or U937, all the subsequent experiments were only performed on PMA-differentiated macrophages [4]; and in all subsequent experiments the same initial number of monocytic cells (1 × 10^6^/mL) was used. After differentiation, macrophages were de-induced in culture medium with reduced FBS (RPMI, 1% FBS, 1% P/S) for 16–18 h. After de-induction, cells were washed with PBS and fresh complete (10% FBS, 1% P/S) RPMI medium was added. Stimulation of PMA-differentiated macrophages under polarizing conditions was performed as described for 48 h (Fig. 1). M1 conditions were LPS (Sigma-Aldrich, Merck) (100 ng/mL) and IFN-γ (Peprotech Inc) (20 ng/mL). M2 conditions were IL-4 (Peprotech Inc) (20 ng/mL) and IL-13 (Peprotech Inc) (20 ng/mL). Control ‘M0’ macrophages were treated with the same volume of PBS in lieu of M1 and M2 stimuli. For gene (RT-qPCR) and protein (flow cytometry/ELISA) expression experiments, samples (cells, cell lysates, cell culture supernatants) were collected from M0/M1/M2 macrophages at the end of the 48 h stimulation period. For assessment of ROS production and phagocytosis activity, M0/M1/M2 macrophages at the end of the 48 h stimulation period were incubated with the fluorescent ROS substrate or with the FITC-labeled latex beads, respectively, for 30 min before acquisition of data.

### 2.6 Gene expression

Total RNA was extracted from lysed cells using affinity columns according to the manufacturer's protocol (Cellco, BR). The quantity and purity of total RNA were determined by UV spectrophotometry and by the absorbance at 260 nm and the ratio 260/280 nm, respectively. 200 ng of total RNA were converted into cDNA using random hexamer primers and synthetic reverse transcriptase in a reaction volume of 20 μL, following the instructions of the supplier (High-Capacity cDNA Synthesis kit, Applied Biosystems, Invitrogen Corp., Foster City, CA, USA). qPCR reactions were performed in a 10 μL total volume reaction, including SYBR Green mastermix (PowerUp SYBR Green, ThermoFisher Scientific), cDNA template, deionized water, and human-specific pre-designed and optimized sets of primers (Table 1). Cycling conditions were empirically optimized in control samples by assessing the melting curve after 45 amplification cycles. Reactions were run on a StepOne Plus qPCR thermocycler (Applied Biosystems, Invitrogen Corp., Foster City, CA, USA). Relative levels of gene expression were determined by the Δ(ΔCt) method using the thermocycler's software and automated detection of the Ct. Expression of GAPDH (no significant change in Ct in the different experimental conditions for both cell lines) was used in the calculations as the normalizing gene.

**Table 1:** Primer sequences used in the assessment of gene expression by RT-qPCR.

Gene	Transcript	Sequence (5′-3′)
***GAPDH***	NM_002046.7	S- ACAACTTTGGTATCGTGGAAGG
		AS-GCCATCACGCCACAGTTT C
***IL10***	NM_000572.3	S - GGCACCCAGTCTGAGAACAG
		AS - ACTCTGCTGAAGGCATCTCG
***TNF***	NM_000594.4	S - GCTGCACTTTGGAGTGATCG
		AS - TCACTCGGGGTTCGAGAAGA
***IL6***	NM_000600.5	S- CCTGAACCTTCCAAAGATGGC
		AS- TTCACCAGGCAAGTCTCCTCA
***ARG1***	NM_000045.4	S- GTGGAAACTTGCATGGACAAC
		AS- AATCCTGGCACATCGGGAATC

### 2.7 Phagocytosis activity

In this experiment PMA-differentiated macrophages from THP-1 and U937 cells in M0, M1 and M2 conditions at the end of the 48 h stimulation period were incubated for 30 min (at 37 °C and 5% CO_2_) with 8 μL of 2.0 μm FITC-latex beads (L4530; Sigma-Aldrich, St Louis, MO, USA) added to 500 μL of complete culture medium. After the 30-min incubation, cells were thoroughly washed with PBS, enzymatically dissociated from the culture substrate (Accutase, BD Biosciences), resuspended in PBS + 2% FBS and analyzed by flow cytometry (BD FACS Verse, BD Biosciences). The experiment was performed in triplicate with acquisition of 10,000 events in each sample.

### 2.8 Production of ROS

CM-H2DCFDA substrate (Thermo Fisher Scientific) (50 ng) was added to cultures of PMA-differentiated macrophages at the end of the 48 h stimulation period (M0, M1, and M2 phenotypes). Following a 30 min incubation at 37 °C in the dark, cells collected by gentle enzymatic dissociation (Accutase, BD Biosciences), washed in PBS and resuspended in PBS + 2% FBS. Emission of fluorescence by ROS-converted substrate was detected using flow cytometry (BD FACS Verse, BD Biosciences). The experiment was performed in triplicate with the acquisition of 10,000 events/sample.

### 2.9 Cytokine secretion

Cell culture supernatants from PMA-differentiated macrophages were collected at the end of the 48 h polarization period (M0, M1, M2 phenotypes). Secretion of cytokines that were not exogenously added to the macrophages during polarization, namely IL-6 (Interleukin-6), and IL-1β (Interleukin-1β), was quantified using sandwich ELISAs, according to the manufacturer's instructions (Peprotech Inc.). The results were normalized to the total protein concentration in the cell culture supernatant samples. Data is derived from three independent experiments performed in triplicate.

### 2.10 Flow cytometry

At the end of the 48 h polarization period, macrophages from either cell line were collected by gentle enzymatic (Accutase, BD Biosciences) detachment from the cell culture substrate, resuspended, counted on a hemocytometer and resuspended in flow cytometry staining buffer (PBS supplemented with 2% FBS) at 1 × 10^6^ cells/mL. After blocking Fc receptors (CD16/CD32 Human Fc Block, BD Biosciences), aliquots of cells were stained for 30 min at room temperature and protected from light with fluorescent conjugated antibodies (CD68-FITC from BD Biosciences and CD163-PE.Cy7 from Biogems) according to the concentrations indicated by the suppliers. Data was acquired on a Cytoflex (Beckman-Coulter) flow cytometer using an automatic compensation matrix generated using flow cytometry beads and the same primary antibodies. A minimum of 10,000 events were acquired for each sample and the experiments were repeated three times independently.

### 2.11 Data analyses

Data obtained from each experiment were analyzed using GraphPad Prism 6.0 (GraphPad Software Ind., San Diego, CA, USA). The purpose of the analysis was to compare the outcomes assessed between macrophage derived from the two monocytic cell lines (THP-1 vs U937) in the same experimental conditions and among the polarizing conditions (M0 vs M1 vs M2) within each cell line (THP-1 or U937). The significance level was set at 95% (p < 0.05) in all analyses.

## 3 Results

### 3.1 M-CSF failed to induce differentiation of U937 cells

**Fig. 2:**
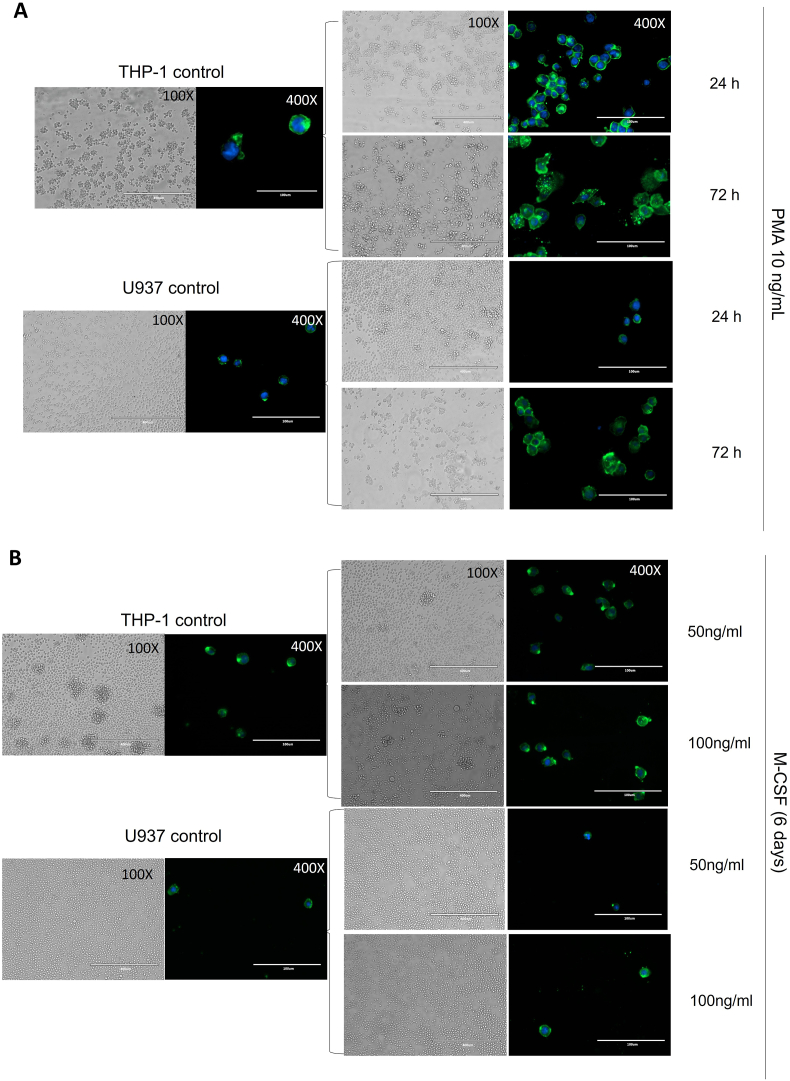
Morphology and substrate adhesion of THP-1 and U937 cells in different differentiation conditions. Representative brightfield and fluorescence images of THP-1 and U937 cells stained with FITC-conjugated phalloidin for visualization of actin filaments of the cytoskeleton. **(A)** PMA stimulation; **(B)** M-CSF stimulation. Images shown were obtained from the central portion of the culture well and are representative of three independent experiments.

After 6 days of stimulation with M-CSF (50 and 100 ng/mL), U937 cells failed to adhere to the culture substrate, as nearly all cells were removed by gentle washing. Similarly, only few THP-1 cells remained attached to the culture substrate at the end of the 6-day differentiation with M-CSF. In the few attached cells there was no clearly visible changes in cell size in comparison with undifferentiated control cells (Fig. 2 B). These results indicate that M-CSF does not induce attachment of either THP-1 or U937 cells to the culture substrate, as a phenotypical trait or macrophage differentiation. Thus, for all subsequent experiments, only PMA was used as macrophage differentiation stimulus. The same standardized differentiation protocol [4] was used to allow for direct comparisons between the biological responses of macrophages derived from either monocytic cell line.

### 3.2 Morphology

#### 3.2.1 THP-1 and U937 cells cluster together in PMA-induced differentiation

Neither monocytic cell line showed morphological changes in comparison to unstimulated controls at 24 h. Despite using the same initial number of monocytic cells, there was comparatively more attached THP-1 than U937 cells at 24 h, which suggests that the efficiency of macrophage differentiation was greater for THP-1 cells in comparison with U937 cells using this protocol. There was a statistically significant increase in cell size of THP-1 and a clearly noticeable (albeit non-statistically significant) increase in size of U937 cells at the end of the differentiation period in comparison with cell sizes after the 24 h stimulation with PMA. This indicates that PMA-induced macrophage differentiation progresses over the 48 h period of resting culture in PMA-free culture medium of the differentiation protocol for both monocytic cell lines. Macrophages derived from both monocytic cell lines maintained a similar ‘clustering’ pattern, organizing in small colony-like formations that were firmly adhered to the culture substrate (Fig. 2 A, Supplementary Fig. 1).

### 3.3 Gene expression

Expression of TNF, IL-6, IL-10, and Arg-1 was differentially regulated by M1 and M2 stimuli in THP-1 and U937-derived PMA-differentiated macrophages.

**Fig. 3:**
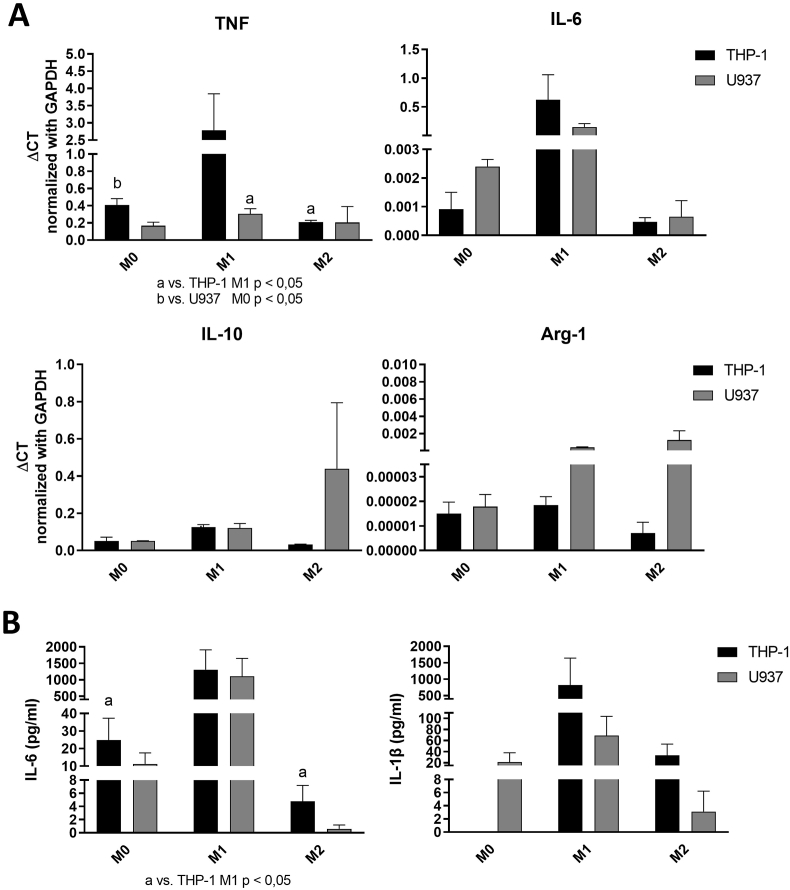
Distinct responsiveness of THP-1- and U937-derived macrophages to M1 and M2-polarizing conditions. THP-1 was more responsive to M1 stimuli and U937 was more responsive to M2 stimuli in gene expression and cytokine secretions analysis**: (A)** Normalized candidate gene expression determined by RT-qPCR in resting, M1 and M2 conditions. (a) p < 0.05 compared with the THP-1 M1 group. (b) p < 0.05 compared with the U937 M0 Group **(B)** Secretion of IL-1β and IL-6 by THP-1 and U937-derived macrophages in resting, M1 and M2 conditions quantified by ELISA. Cytokine concentrations were normalized to the concentration of total protein in the culture supernatants. (a) p < 0.05 compared with the THP1 M1 group. In **(A)** and **(B)** bars represent average and vertical lines the standard deviation of three independent experiments assessed in duplicate.

In general, THP-1-derived macrophages were more responsive to M1 stimulation and U937-derived macrophages were more responsive to M2 stimulation. TNF was induced in M1 conditions only in THP-1-derived macrophages. Moreover, fold change in IL-6 expression induced by M1 conditions was also greater in THP-1-derived macrophages. On the other hand, IL-10 expression was induced by M2 stimuli only U937-derived macrophages, which also had increased regulation of Arg-1 expression in both M1 and M2 conditions. (Fig. 3 A).

### 3.4 Cytokine secretion

Regulation of IL-1β and IL-6 in M1 and M2 conditions is similar in macrophages from both THP-1- and U937-derived macrophages.

Regulation of IL-6 production was similar in macrophages derived from both cell lines: M1 conditions greatly increase IL-6 secretion, whereas M2 stimuli decreased IL-6 production below levels in M0 macrophages. Regulation of IL-1β production was similar (increased in M1, reduced in M2 conditions) for U937-derived macrophages, whereas in THP-1-derived macrophages secretion of IL-1β in M2 conditions was much lower than under M1 stimuli, but still greater than in M0 conditions, which had no detectable production of this cytokine. Overall, production of these candidate pro-inflammatory cytokines was more pronounced in THP-1-derived macrophages. (Fig. 3 B).

### 3.5 Flow cytometry

**Fig. 4:**
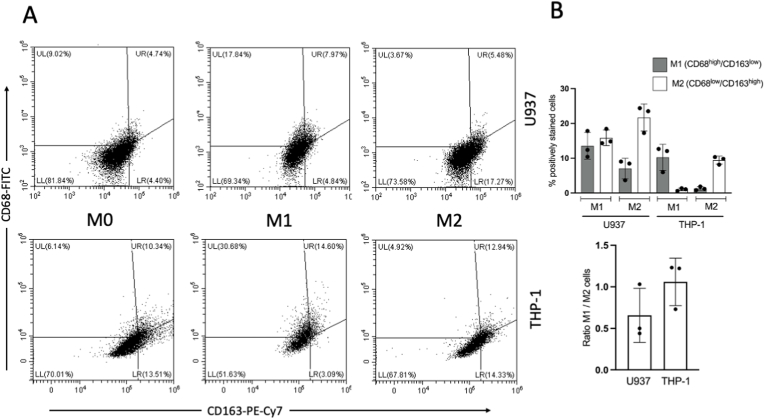
**–** Phenotypical skewing of U937- and THP-1-derived macrophages under M1 and M2 conditions. At the end of the differentiation/polarization period, macrophages derived from U937, and THP-1 cells were collected by gentle enzymatic dissociation and stained with primary fluorophore-conjugated antibodies for CD68 and CD163. **(A)** Representative dot-plots of phenotype assessment by flow cytometry and the quadrant gates for M1 (Upper Left/UL, CD68^high^/CD163^low^) and M2 (Lower Right/LR, CD68^low^/CD163^high^). **(B)** Graphs of percentage of cells classified as M1 or M2 according to the cell type and experimental conditions (M1: 100 ng/mL LPS + 20 ng/mL IFN-γ; M2: 20 ng/mL IL-4 + 20 ng/mL IL-13). Note that U937-derived macrophages are more responsive than THP-1-derived macrophages in general and in M1 conditions there is a considerable proportion of cells classified as M2. Lower graph presents the ratio of the percentage of macrophages classified as M1 and M2 according to the cell type. Ratios below 1.0 indicate a skewing towards M2, and conversely ratios above 1.0 indicate a skewing towards M1. Data derived from three independent experiments with each cell line and a minimum of 10,000 events acquired for each sample.

At the end of the differentiation/polarization period, unstimulated (M0) U937 cells were slightly skewed to the M2 phenotype (greater percentage of CD163+ cells) in comparison to U937 cells. THP-1 cells were also more responsive to M1 stimuli as the percentage of CD68^+^ cells increased nearly 4-fold (versus less than 2-fold for U937 cells). Macrophages derived from both cell lines behave as expected according to the nature (M1/M2) of the stimuli; however, THP-1 cells skewed more towards M1 end of the polarization spectrum and conversely U937 cells skewed more towards M2 end of the spectrum (Fig. 4).

### 3.6 Phagocytosis

#### 3.6.1 THP-1-derived macrophages have greater phagocytic activity

**Fig. 5:**
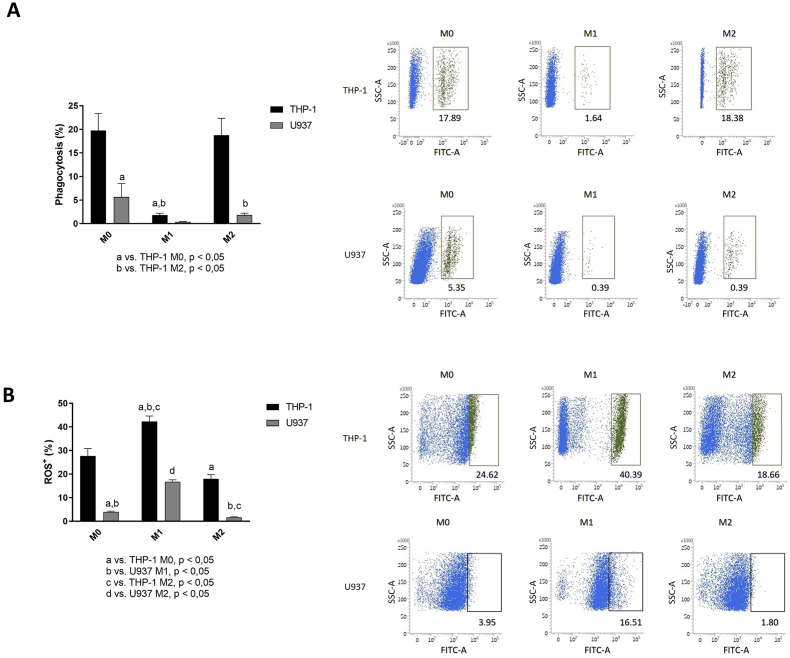
Functional differences of THP-1- and U937-derived macrophages: **(A)** Phagocytic activity of THP-1-derived macrophages is greater than that of U937-derived macrophages. In M1 conditions, phagocytosis is markedly reduced in macrophages derived from both cell lines, and M2 conditions only reduced phagocytosis in U937-derived macrophages. (a) p < 0.05 compared with the THP-1 M0 group. (b) p < 0.05 compared with the THP-1 M2 group. The dot-plots show the percentage cells realize phagocytosis. The right gate on y-axis corresponds to initial phagocytosis and the left gate represents phagocytosis in final stage. The Dot-plots represented the M0, M1 and M2 phenotype corresponded to THP-1 and U937 line, respectively (**B)** Production of ROS is also higher in THP-1-derived macrophages and n macrophages derived from both THP-1 and U937, M1 conditions increase and M2 conditions decrease the percentage of ROS-producing cells. (a p < 0.05 compared with the THP-1 M0 group. (b) p < 0.05 compared with the U937 M1 group. (c) p < 0.05 compared with the THP-1 M2 group. (d) p < 0.05 compared with the U937 M2 group. The Dot-plots represented the M0, M1 and M2 phenotype corresponded to THP-1 and U937 line, respectively. In both **(A)** and **(B)**, bars represent average and vertical lines the standard deviation of three independent experiments, each acquiring 10,000 events. Representative dot-plots with gates indicating the percentage of phagocytosing **(A)** or ROS-producing **(B)** cells, according to the monocytic precursor cell line and conditions.

Phagocytosis by THP-1-drived macrophages cells was markedly greater than that of U937-derived macrophages in all phenotypical conditions. M1 stimuli significantly reduced phagocytic activity of THP-1-derived macrophages, whereas M2 conditions had no effect in comparison with M0 condition. U937-derived macrophages had the greatest phagocytic activity in M0 condition, whereas phagocytosis was nearly abrogated in M1 conditions and significantly decreased by M2 stimuli. (Fig. 5 A).

### 3.7 Production of ROS

#### 3.7.1 THP-1-derived macrophages produce more ROS

The percentage of ROS-producing cells was greater in THP-1-derived macrophages than in U937-derived macrophages across all phenotypical conditions. In macrophages derived from both cell lines M1 conditions increased and M2 stimuli reduced the percentage of ROS-producing cells. (Fig. 5 B).

## 4 Discussion

Macrophages are terminally differentiated cells and their precise origin is still currently under debate, as they may have an embryonic precursor generating a population of resident macrophages and may also differentiate from circulating monocytes of myeloid origin [25,32]. Regardless, the isolation of primary macrophages directly from tissues is technically challenging and limited in yield. Ontologically, it may be argued that most macrophages are derived from marrow-derived circulating monocytes, which are experimentally used as precursor cells that are induced to differentiate into macrophages in in vitro studies. Established immortalized monocytic cell lines are frequently used as a source of macrophages, which could facilitate comparisons among studies using standardized methods because of the greater homogeneity in cell response. In addition, the virtually unlimited supply of non-senescent, immortalized cell lines solves the difficulties associated with the use of primary monocytes from PBMC (2–10% of PBMC are monocytes) such as ethical implications in collecting whole blood from volunteer donors, the need to separate mononuclear cells and sort monocytes; and inter- and intra-individual variability associated with differences in the genetic/epigenetic makeup and basal phenotype conditions related with gender, age, systemic conditions, and the use of medications. However, the choice of an established monocyte precursor cell line is often unjustified biologically in the studies; and the methods used to differentiate, induce/activate and assess the phenotype are highly variable. Moreover, established monocytic cell lines are usually derived from patients with hematological neoplasies, and thus their responses may deviate markedly from normal cells.

THP-1 and U937 are two widely used established human monocytic cell lines considered to be capable of mimicking some responses of primary monocytes and macrophages [1,31]. This study compares the responses of THP-1 and U937 cell lines in their ability to differentiate into macrophages and on common cellular responses of macrophages, including gene expression, phenotypical polarization, production of ROS and phagocytosis.

It is important to note that there is a plethora of macrophage differentiation protocols reported in the literature and even polarizing conditions may vary regarding the nature, concentration and period of simulation. This heterogeneity in experimental conditions certainly account for a considerable portion of contrasting observations in the literature regarding the biological response of macrophages derived from either monocytic cell line. In this study we used standardized protocols for macrophage differentiation and phenotypical polarization to allow for a direct comparison in biological responses of macrophages derived from THP-1 and U937 monocytic cells.

Overall, both cell lines can differentiate into macrophages (based on changes in cell size and attachment to culture substrate) but their responses to M1 and M2 polarizing stimuli are distinct: THP-1 cells are more responsive to M1 conditions and U937 cells are shifted towards the M2 phenotype.

Granulocyte–macrophage colony stimulating factor (GM-CSF) and macrophage colony stimulating factor (M-CSF) are major endogenous growth factors implicated in macrophage differentiation from monocytes [15]. Notably, stimulation with M-CSF in concentrations and periods sufficient to induce differentiation of primary monocytes did not induce macrophage differentiation of either THP-1 and U937 cells. Aldo et al. also reported on the lack of responsiveness to M-CSF by THP-1 cells, as it did not increase expression of CD14 [1]. This may be associated with expression/functionality of M-CSF receptor by these cell lines, although there is a report indicating c-Fms gene and M-CSFR protein expression and activation (Tyrosine phosphorylation) in IL-12-stimulated THP-1 and U937 cells [22]. Other possibilities include genetic drifting with changes in phenotype which occurs with widely-distributed cell lines, differences in culture conditions such as cell density that can affect responsiveness to external stimuli [1], and accidental contamination with other cell lines. According to information on American Type Culture Collection's (ATCC) website (https://www.atcc.org/products/crl-1593.2), in 1994 PCR and cytogenetic analyses showed that a number of stocks of U937 were contaminated with the human myeloid leukemia cell line K-562, which prompted the temporary discontinuation of its distribution. In this study, we used recently acquired stocks of both THP-1 and U937 cells, the latter from a new stock (ATCC CRL-1593.2) that was tested and verified to be free of second contaminant subpopulations.

Differentiation protocol with PMA was used as recently described [4] to obtain macrophages in a less-activated state, consistent with an M0 phenotype. Using this protocol, both THP-1 and U937 cells adhered to the culture substrate after the initial 24 h, but there was no marked difference in cell morphology, which was clearly noticeable as increased size for both cell lines after 72 h, which is also reported by Ref. [1]. Importantly, expression of candidate pro-inflammatory genes in the absence of stimulation was lower than that after M1 polarization, but higher than that observed after M2 stimulation, suggesting that this differentiation protocol yielded cells that were highly responsive to stimulation in either direction of the macrophage phenotype spectrum. At the end of the differentiation protocol, THP-1 cells had a more irregular morphology, which is associated with pro-inflammatory/M1 macrophages; whereas U937 cells were more uniformly round, a morphology associated with an M2 phenotypical profile [33,37]. As M1 and M2 stimuli, we used 100 ng/mL LPS (100 ng/mL) + IFN-γ (20 ng/mL) and IL-4 (20 ng/mL) + IL-13 (20 ng/mL), respectively, which are commonly reported in the literature [4,28,38], but certainly not ‘consensual’. Differences in the nature, concentration and period of stimulation of ‘M1’ and ‘M2’ conditions are frequently noted in the literature, which may also account for contrasting observations reported in published studies.

Interestingly, subsequent experiments supported this morphological association with macrophage phenotype, as THP-1 cells skewed more to an M1 phenotype (increased production of ROS and expression of pro-inflammatory cytokines), whereas U937 cells were shifted towards the M2 phenotype, with reduced expression and secretion of pro-inflammatory cytokines, increased expression of IL-10 and lower production of ROS, which is associated with microbicidal activity, reported as more pronounced in M1 macrophages [39].

Notably, this trend of M1 skewing for THP-1-derived macrophages and M2-skewing for U937-derived macrophages was consistent for both gene (RT-qPCR) and protein expression (ELISA and flow cytometry). This difference may be related with the uncharacteristic TLR4/NF-kB pathway in U937 cells, which affects their response to LPS, one of the exogenous agonists used for simulation of M1 conditions [30]. However, it is important to consider that the literature on the biology of monocyte-derived macrophages is diverse and often controversial. In this context, there is evidence that THP-1 cells upregulated expression of MRC1 (CD206) and of hemoglobin scavenger receptor (CD163) genes in M2 conditions [16,29]; however the upregulation of CD163 by these cells have been more strongly associated with exogenous stimulation with IL-10 in comparison to IL-4 as M2-inducing condition [21]. In contrast, another study [9] reported that THP-1-derived macrophages stimulated with IL-10 did not increase expression of membrane-bound CD163. This illustrates the complex biology of macrophages and the heterogeneous information available in the literature on these monocytic cell lines.

Phagocytic activity in vivo is reported to be more pronounced in M2 macrophages, [8,14,35]; and indeed, under M1 conditions, both THP-1- and U937-derived macrophages had a significant decrease in phagocytosis. However, phagocytosis may be mediated by distinct receptors in M1 (FcR, Complement receptors) or M2 (non-opsonin receptors: mannose receptor, DC-SIGN, integrins) conditions [18]. Nevertheless, M2 condition did not enhance phagocytosis by macrophages derived from either monocytic cell line; which is contrary to the findings of [34]; who reported a reduction in phagocytosis by THP-1-derived macrophages under the same M2 conditions and an increase in phagocytosis associated with the same M1 conditions. In fact, for U937-derived macrophages M2 stimuli reduced phagocytosis in comparison to unstimulated (M0) conditions. Notably, the M1-skewed THP-1-derived macrophages had significantly greater phagocytic activity than U937-derived macrophages.

Interestingly [18], reported differences in phagocytitic activity of THP-1-derived macrophages according to the differentiation stage and influenced by the phenotype. Contrasts between our results and previous studies using the same monocytic cell lines are likely to be associated with the differentiation protocol, including concentrations of PMA, period of treatment and the inclusion of a ‘resting’ period after differentiation. Prasad et al. showed that differentiation of U937 monocytes with 100 ng/mL of PMA induced increased ROS production [27]. Kuno et al. showed that phagocytosis by the U937-derived macrophages was inversely related with the concentration of PMA used in their differentiation, with lower phagocytic activity associated with higher concentrations of PMA [17]. Other important details of the protocols, such as time of incubation with latex beads in phagocytosis assays may account for some contrasting findings. Collectively, this information highlights the importance of considering the differentiation and other experimental protocols as important variables that may significantly affect the functional outcome of the differentiated macrophages.

It is important to acknowledge that M1 pro-inflammatory and M2 anti-inflammatory M2 phenotypes are extremes in a spectrum that are not absolute or mutually exclusive, and macrophages assume a phenotype in this spectrum that usually skews toward either extreme, meaning that the predominant genes expressed and activity of biological functions conform to the phenotype in one end of the spectrum, but some characteristics of the opposite end of the phenotype spectrum may remain [13]. Similarly, the cytokines expressed are not unique to each profile and can lead to different responses depending on concentration, timing of exposure and context (other cues in the microenvironment). It has been observed that exposure of M2 macrophages to the cytokine IL-6, usually considered as pro-inflammatory, may further support this phenotype instead of skewing macrophages to the M1 phenotype [2].

It is important to consider the limitations and note the main purpose of this study. We have used a single method/procedure for macrophage differentiation and polarization; which albeit limiting for the applicability of the results, is essential for a proper direct comparison of the biological response of macrophages derived from each monocytic cell line. Also, we have used only a single marker for flow cytometry assessments of M1 and M2 phenotypes. The scope of the manuscript was to compare the biological response of macrophages derived from the two most used monocytic cell lines, which may help in planning experiments and interpreting data reported in the literature using these cell lines.

Overall, these of this study indicate that, under the standardized PMA-induced differentiation conditions, THP-1-derived macrophages are more responsive to pro-inflammatory (M1) stimuli, whereas U937-derived macrophages are skewed towards the M2/alternative phenotype. This information is useful in planning studies using these monocytic cell lines that emphasize a specific pro- or anti-inflammatory phenotype or a specific biological function of macrophages.

## 5 Conclusion

Our results show that the both THP-1 and U937 human monocytic cell lines can differentiate into macrophages and mimic the responses of primary macrophages. Also under the same standardized PMA differentiation conditions, macrophages derived from each cell line have distinct biological responses to the same stimuli, which makes them not ‘interchangeable’ as in vitro model of macrophage precursor cells. Thus, even under the same differentiation conditions, the choice of monocytic precursor cell should be based on the biological responses, goals, and biological context of interest.

## Funding information

This work was supported by the Coordination of Superior Level Staff Improvement (10.13039/501100002322CAPES- 001) São Paulo Research Foundation - 10.13039/501100001807FAPESP grant# 2018/24240-4 to CRJ.

## Declaration of competing interest

The authors declare that they have no known competing financial interests or personal relationships that could have appeared to influence the work reported in this paper.

## Data Availability

Data will be made available on request.

## Abbreviations

cDNA: complementary DNA DAPI, 4′,6-diamidino-2-phenylindole
DAMPs: Damage Associated Molecular Patterns- DAMPs
EDTA: ethylenediaminetetraacetic acid
ELISA: enzyme-linked immunosorbent assay
FBS: fetal bovine serum
GM-CSF: Granulocyte–macrophage colony stimulating factor
IFNγ: interferon-γ
IL1β: interleukin 1β
IL-4: interleukin-4
IL-6: interleukin 6
IL-10: interleukin 10
IL-12: interleukin 12
IL-13: interleukin 13
LPS: lipopolysaccharide
M-CSF: macrophage colony stimulating factor
MAMPs: Microbe Associated Molecular Patterns
P/S: penicillin and streptomycin
PBS: phosphate buffered saline
PMA: phorbol 12-myristate 13-acetate
PBMC: peripheral blood mononuclear cells
RT-qPCR: real-time quantitative polymerase chain reaction ROS, reactive oxygen species
RPMI: Roswell Park Memorial Institute
TGFβ1: transforming growth factor β
TLP: toll-like-receptor
TNFα: tumor necrosis factor α

## Appendix A Supplementary data

Figs1 Area and perimeter of the U937- and THP-1-derived macrophages after 24 and 72 h of PMA stimulation. Digital brightfield images were obtained at 40X magnification in three random fields of each well. A trained examiner who was blind to the cell line and experimental period performed the measurements of area and perimeter on all cells present in the microscopic field images using ImageJ software. Data shown is the average and standard deviation of three independent experiments, each assessing all cells in three random microscopic fields. Figs1
