# Supplementary figures and images for: Comparison of monocytic cell lines U937 and THP-1 as macrophage models for in vitro studies
Source: Biochem Biophys Rep. 2022 Nov 18;32:101383. doi: 10.1016/j.bbrep.2022.101383 (PMC9677084; doi:10.1016/j.bbrep.2022.101383)

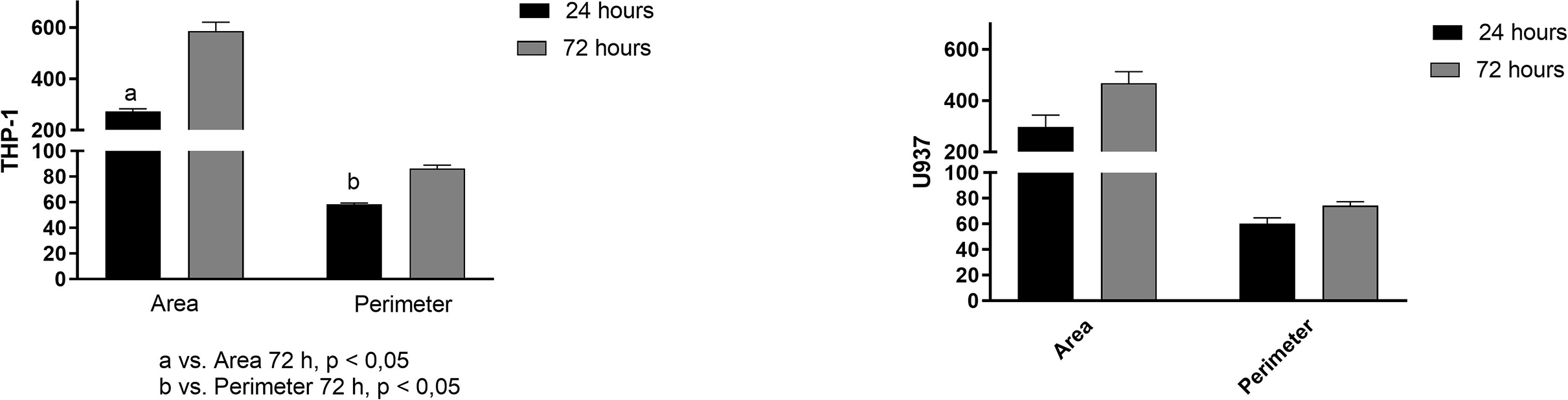

Supplement: Figs1 — Area and perimeter of the U937- and THP-1-derived macrophages after 24 and 72 h of PMA stimulation. Digital brightfield images were obtained at 40X magnification in three random fields of each well. A trained examiner who was blind to the cell line and experimental period performed the measurements of area and perimeter on all cells present in the microscopic field images using ImageJ software. Data shown is the average and standard deviation of three independent experiments, each assessing all cells in three random microscopic fields. [file mmcfigs1.jpg]
